# Combinative Scouring, Bleaching, and Cationization Pretreatment of Greige Knitted Cotton Fabrics for Facilely Achieving Salt-Free Reactive Dyeing

**DOI:** 10.3390/molecules22122235

**Published:** 2017-12-18

**Authors:** Wei Ma, Kezhan Shen, Nan Xiang, Shufen Zhang

**Affiliations:** State Key Laboratory of Fine Chemicals, Dalian University of Technology, Dalian 116023, China; Shenkz@mail.dlut.edu.cn (K.S.); xiangnan92@163.com (N.X.)

**Keywords:** combinative pretreatment, cationic cotton, salt-free dyeing, reactive dyes

## Abstract

In order to facilely achieve pretreatment and salt-free dyeing of greige knitted cotton fabrics, a combinative scouring, bleaching, and cationization pretreatment of the fabrics is designed in this study. The fabrics are first treated in a bath containing commercial scouring and bleaching agents, and then glycidyltrimethylammonium chloride (GTA) is directly added into the bath to achieve cationization of the fabrics. Utilization of the alkaline and high-temperature conditions in scouring and bleaching process, cationization can facilely proceed in a short time. Optimal pretreatment conditions are as follows: greige knitted cotton fabrics are treated in a bath containing 4 g/L scouring agent and 6 g/L 30% hydrogen peroxide at 90 °C for 60 min, and then 30 g/L GTA and 3 g/L sodium hydroxide are added in the bath for another 15 min treatment. Fiber performances, including whiteness, water absorptivity, diffusion time, and capillary effect, are tested and evaluated. X-ray diffraction analysis, surface morphology, and thermal analysis of the pretreated cotton are also investigated and compared with that treated only with scouring and bleaching agents. Much higher dye fixation and color yield could be realized on the pretreated cotton in salt-free reactive dyeing. Colorimetric properties of the dyes are studied and good colorfastness of the dyes on the cationic fabrics are obtained. All of the above results show promising prospects of this combinative pretreatment in real application.

## 1. Introduction

Cotton cellulose has splendid properties, such as high moisture absorbency, being comfortable to wear, and easy to dye. For these reasons, cotton textiles are very popular in our life and the textile industry is predominantly cotton-based [1,2,3,4,5]. Cotton is composed of 90–96% cellulose based on the weight of the fibers. The impurities in the fibers range from 4% to 10%. These non-cellulosic components are waxes, pectins, and proteins, and are mainly found in the cuticle layer and the primary wall, which are the outermost layers of the cotton fibers [6,7,8]. In addition to those mentioned above, resins, pigments, and hemicellulose also exists in greige cotton fabric [9,10,11]. The yellowish or brown coloration of the cotton fibers is related to the protoplasmic residues of protein and the flavones pigments of cotton flowers [12,13].

Scouring process of the greige cotton fabric removes these impurities for better bleaching and dyeing properties. With the exception of pigments which removed by alkali treatment in a process known as scouring. Scouring, in common practice, involves boiling the cotton in sodium hydroxide (2–5%) for about 1 h [14,15,16]. In order to promote scouring agent penetration in the cotton fibers and emulsify the wax on cotton, surfactants are added during the scouring process. After scouring, water absorbency is improved, the appearance of cotton fabrics becomes clean and soft. However, the natural pigment has not been removed in the scouring process, and the whiteness does not meet the requirement. The bleaching process can effectively improve the whiteness. Hydrogen peroxide is an environmentally safe bleaching agent for cotton fabrics. However, bleaching of cotton-based fabrics with hydrogen peroxide requires an alkaline medium (normally NaOH), stabilizer, and either high temperatures or a long dwell time [17,18], which results in high energy consumption and even gives rise to some fiber damage. Additionally, it is essential to neutralize the bleach solution and rinse the fabric with copious amounts of water when the bleaching process is complete [19]. There is still a large amount of alkali in the residue liquid and these residue liquids cannot be reused. Wastewater treatment processes will be more difficult because of the alkaline residue.

Cotton fabrics are commonly dyed with reactive dyes, which are very popular because of their brilliance, wide range of hues, and excellent fastness properties. As cotton fibers show a slightly negative charge and reactive dyes are anionic in solution, in exhaust dyeing, 30–150 g/L electrolytes, such as sodium chloride or sodium sulfate, need to be added for dye exhaustion [20]. The release of effluent containing large quantities of salt will cause pollution of rivers and streams and upset the biological equilibrium [21,22]. Increasing the substantivity of reactive dyes to cotton can reduce or even eliminate salt addition. In this regard, the surface modification of cellulosic fabrics using cationic compounds has attracted much attention for solving the problem [3,4,23,24,25].

**Figure 1:**
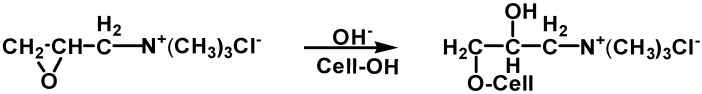
Reaction of GTA with cellulose.

Cotton is cationized through the reaction of various types of substituted amino compounds [24]. The introduction of the amino groups renders the fibers’ cationic property, increasing their affinity for anionic dyes [26,27]. Glycidyltrimethylammonium chloride (GTA) has been used to cationize cotton and effectively improve dye uptake [26,27]. It is a highly-active small molecular cationic reagent, and its reaction with the fibers usually proceeds with addition of sodium hydroxide (see Figure 1).

**Figure 2:**
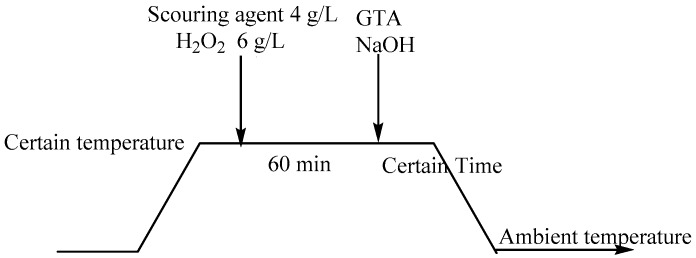
Schematic of the combinative pretreatment of scouring, bleaching, and cationization.

**Figure 3:**
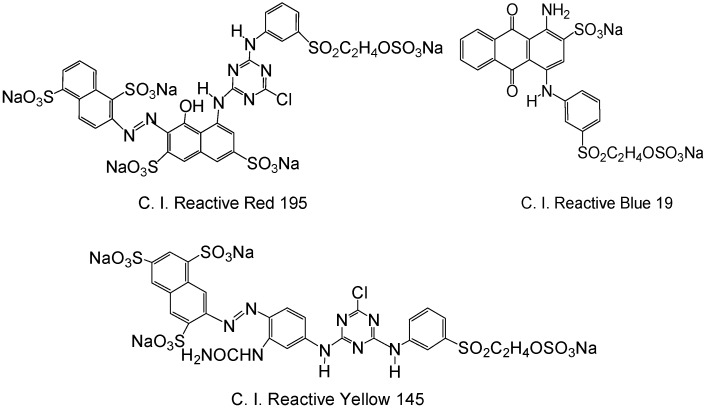
Structures of the reactive dyes used in this study.

It is found that the scouring, bleaching, and cationization of greige cotton fibers are all under alkaline conditions. If these procedures are combined into one, alkali dosage, treatment time, consumed energy and water, and overall cost will be greatly reduced, and the process will be much more convenient and beneficial for achieving pretreatment and salt-free reactive dyeing. In previous studies, scouring and bleaching treatment can be combined [28]; however, their combination with cationization has not been reported. In this work, a new combinative approach for fabric treatment and cationization is postulated. Greige knitted cotton fabrics are chosen for investigation due to their wide application in exhaust dyeing, in which a large amount of inorganic salt, including sodium sulfate or sodium chloride is commonly used. With the proposed combinative pretreatment approach, the fabrics were scoured with commercial agent, bleached with H_2_O_2_ and catioinized with glycidyltrimethylammonium chloride in one alkaline pretreatment bath (See Figure 2). Through investigation, it was found GTA could not be added at the beginning of scouring and bleaching, as the conditions employed in the process are severe and show adverse effects for cationization. Satisfactory results could be obtained when scouring and bleaching were first carried out in one bath and at the end of the process, GTA was added in the bath for a short time to cationize the cotton. The project of this study is to optimize the pretreatment conditions, examine the fibers properties including whiteness, water absorptivity, diffusion time, capillary effect, surface morphology, and thermal properties, and investigate the dyeing performance of C. I. Reactive Red 195, C. I. Reactive Yellow 145, and C. I. Reactive Blue 19, including dye fixation, color yield, and colorimetric and colorfastness properties. The structure formula of the reactive dyes is shown in Figure 3.

## 2. Results and Discussion

### 2.1. Effect of Scouring and Bleaching Pretreatment

In order to investigate the properties of cotton fabrics after scouring and bleaching, whiteness, capillary effect, and water absorptivity were measured. Table 1 gives the results of whiteness of the cotton scoured for 30, 45, 60, and 75 min. Wherein, the concentrations of scouring agent and hydrogen peroxide were 4 g/L and 6 g/L, respectively, and the reaction temperature used was 90 °C. It showed whiteness increased from 68.07 to 77.04 when scouring time extended from 30 to 60 min. However, whiteness experienced a little decrease to 76.54 when scouring time prolonged to 75 min. Thus, we prefer to choose 60 min as optimal scouring and bleaching time and the obtained whiteness is in line with the national standard of 75 ± 5 stipulated in GBT 25813-2010. Water absorptivity of the scoured cotton reached 238% which also meets the standard in GBT 21655.1-2008. In addition, the average diffusion time of water droplet was 2.48 s, which satisfied the requirement of less than 3 s in GBT 21655.1-2008. After pretreatment, capillary effect of cotton fibers in warp and weft are 10.4 cm/15 min and 10.1 cm/15 min, respectively which both meet the requirement in the national standard.

### 2.2. Optimization of the Cationization Conditions in Combinative Pretreatment

**Figure 4:**
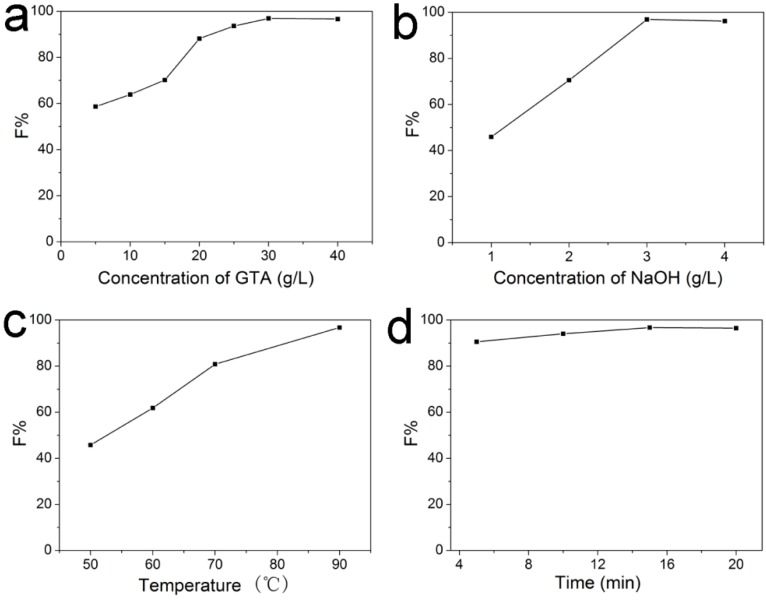
Effect of GTA concentration (**a**); NaOH concentration (**b**); exhaust temperature (**c**); and time (**d**) on F% of C. I. Reactive Red 195 on cationic cotton.

Since GTA was added in the late stage of scouring and bleaching processes, cationization of cotton may be affected by the residue scouring and bleaching agents. Thus, in this study, cationization conditions were optimized for achieving high dye fixation, the fiber properties were also tested to see whether they have reached the standard requirements or not. With all the results, the pretreatment method can be evaluated comprehensively. In this study, the optimal conditions were determined by examining the influence of GTA and alkali dosage, temperature and time on dye fixation (F%). C. I. Reactive Red 195 was employed as the model dye for investigation and results are shown in Figure 4a–d.

#### 2.2.1. Effect of GTA Concentration on F% of C. I. Reactive Red 195 on Cationic Cotton

**Figure 5:**
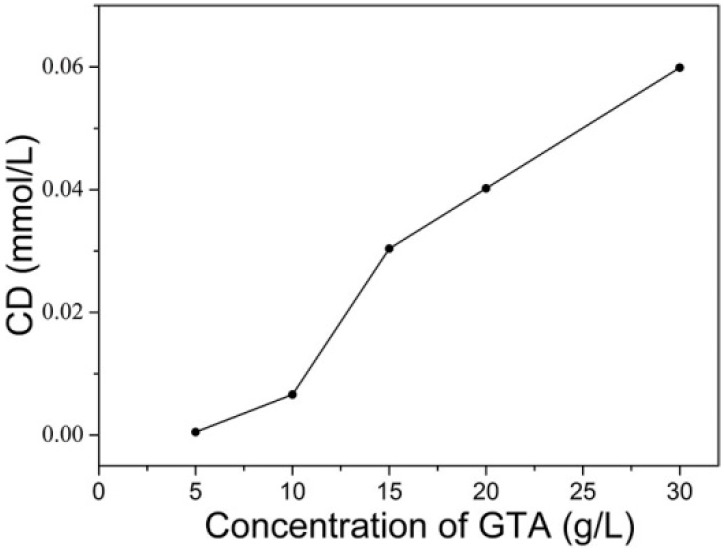
Cationization degree (CD) of modified cotton with different concentrations of GTA.

The effect of the concentration of GTA was first investigated and the results were presented in Figure 4a. Wherein the concentration of sodium hydroxide was 3 g/L, and the bath temperature and reaction time were 90 °C and 15 min for cationization, respectively. Figure 4a shows with the increase of GTA concentration from 5 g/L to 30 g/L, F% of C. I. Reactive Red 195 in the absence of salt increased from 58.66% to 96.74%, indicating that cationization efficiently supplies increased cationic sites on cotton with increasing cationic reagent concentration. In addition, cationization degree of the modified cotton with different concentration of GTA was investigated and showed in Figure 5. Figure 5 presents that cationization degree of the modified cotton improved to 0.0599 mmoL/g from 0.0005 mmoL/g as concentration of GTA increased from 5 g/L to 30 g/L, which demonstrated that the amount of GTA grafted onto cotton fibers increased with increasing concentration of GTA. This result is corresponding to the improvement of the dye fixation. Therefore, addition of high concentration of cationizing reagent could achieve high cationization degree of cotton, as well as high dye exhaustion and the percentage of the total dye fixed on the cationic cotton. This also demonstrates that the reaction of GTA with cotton can proceed well in the scouring and bleaching bath. No improvement in F% was found when the concentration of GTA further increased, revealing the cationic sites are enough for dye exhaustion at 30 g/L of GTA. 

#### 2.2.2. Effect of NaOH Concentration on F% of C. I. Reactive Red 195 on Cationic Cotton

Alkaline condition is required to activate the cotton during cationization. Although the scouring and bleaching bath contains some alkali, it is found that the condition is slightly insufficient for effective cationization of cotton for further salt-free reactive dyeing. A small amount of sodium hydroxide still need be added. The effect of concentration of NaOH on F% of C. I. Reactive Red 195 was examined and the results were shown in Figure 4b. GTA concentration of 30 g/L, the bath temperature of 90 °C and exhaustion time of 15 min were used in the investigation. It shows when the concentration of NaOH was 1, 2, 3, and 4 g/L, the dye fixation was 45.94%, 70.53%, 96.74%, and 96.18%, respectively. It presents that F% of C. I. Reactive Red 195 did not increase when the NaOH dosage increased from 3 to 4 g/L. Therefore, we prefer to choose 3 g/L as the optimal concentration of NaOH for salt-free dyeing of C. I. Reactive Red 195.

#### 2.2.3. Effect of Cationization Temperature on F% of C. I. Reactive Red 195 on Cationic Cotton

The cationization temperature from 50 °C to 90 °C was studied, and 30 g/L GTA, 3 g/L NaOH and 15 min reaction time were used in this study. The results were shown in Figure 4c. It was evident from the data that in this process, with the increase of the temperature, dye fixation increased distinctly. When the baking temperature increased from 50 °C to 90 °C, F% of C. I. Reactive Red 195 increased from 45.74% to 96.74%. Cationization of cotton with GTA is an etherification reaction, increasing the temperature is beneficial for grafting more cationic groups on cotton. As the cationization is much effective under 90 °C and scouring and bleaching processes are both carried out under 90 °C, we chose this temperature for cationization aiming at achieving both high dye fixation in the absence of salt and convenience for the whole combinative pretreatment.

#### 2.2.4. Effect of Cationization Time on F% of C. I. Reactive Red 195 on Cationic Cotton

Cationization time was set to be 5, 10, 15 and 20 min for investigation, the obtained fixation of C. I. Reactive Red 195 achieved 90.55%, 94.05%, 96.74%, and 96.46%, respectively (See Figure 4d). It can be seen that all dye fixation reached 90% without addition of inorganic salt, and 15 min is good for cationization in this study to gain enough cationic sites for achieving a much high dye fixation.

Based on the above investigations, it can be concluded that the most suitable cationization condition in the combinative pretreatment of cotton for salt-free dyeing of C. I. Reactive 195 is as follows: GTA concentration is 30 g/L, NaOH concentration is 3 g/L, reaction temperature is 90 °C and time is 15 min.

### 2.3. Evaluation of Fiber Properties after Pretreatment

Cationization of cotton may affect the structure of the fibers which will further affect the wearability of it. Therefore, a good preparation method for cationic cotton fibers should not influence their structural properties. In this section, X-ray diffraction and scanning electron microscopy were used to examine the physical properties of the pretreated fibers.

#### 2.3.1. X-ray Diffraction Analysis (XRD)

**Figure 6:**
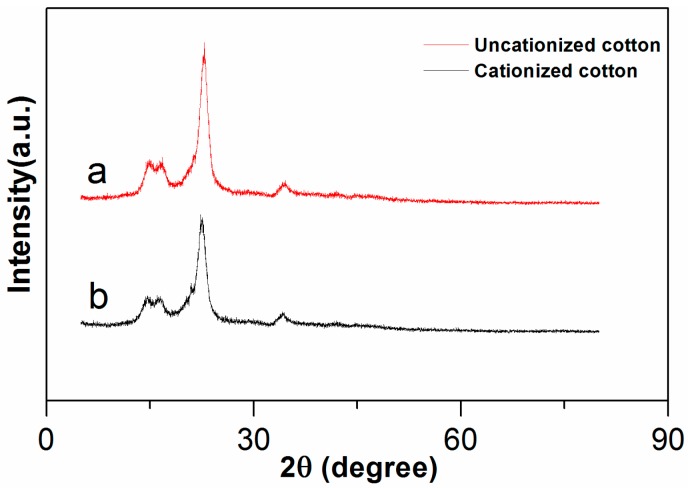
X-ray diffraction patterns of uncationied (**a**) and cationized cotton fibers (**b**).

Chemical modification of cotton fibers may change the crystal form and crystallinity of the fibers, so the X-ray diffraction spectra of the cotton fiber after combinative pretreatment and the control one only after scouring and bleaching steps were measured and compared (as shown in Figure 6). The results showed that the X-ray spectra of the fibers before and after cationization are almost the same, a typical diffraction peak existed at 2 h = 18.6° in both Figure 5a,b. It demonstrated that cationization under optimal conditions occurred just on the surface of the fiber, which had no effect on its crystal structure.

#### 2.3.2. Surface Morphology

**Figure 7:**
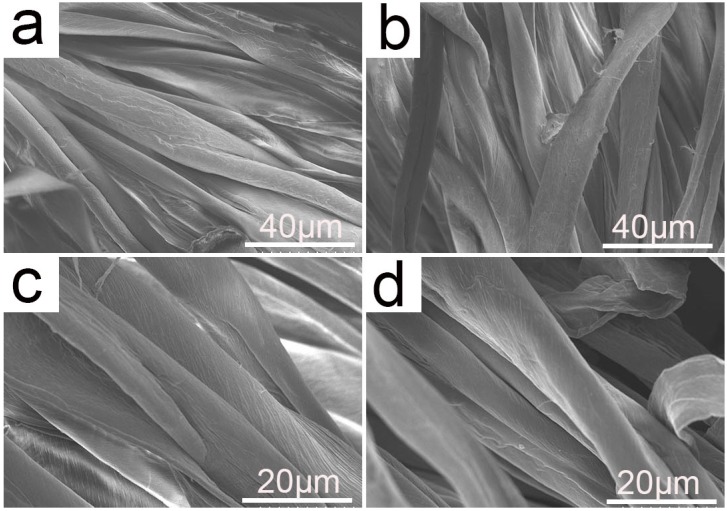
SEM photos of the uncationized (**a**,**c**) and cationized cotton fibers (**b**,**d**).

In this study, scanning electron microscope (SEM) was employed to examine the surface structure of the prepared cationic cotton fibers at the micro-level, which could estimate the influence of cationization process on the fibers. Figure 7a,c shows a SEM photo of the fibers being scoured and bleached and Figure 7b,d is that of being scoured, bleached, and cationized. Based on the photos, although the surface of the cationic fibers was a little rougher compared with that of the uncationized one, no distinct change could be detected between them. As the extent of cationization was small under the selected conditions, the physical structure of the cotton was almost not influenced and the obtained cationic cotton was suitable for dyeing application.

#### 2.3.3. Determination of Thermal Properties

**Figure 8:**
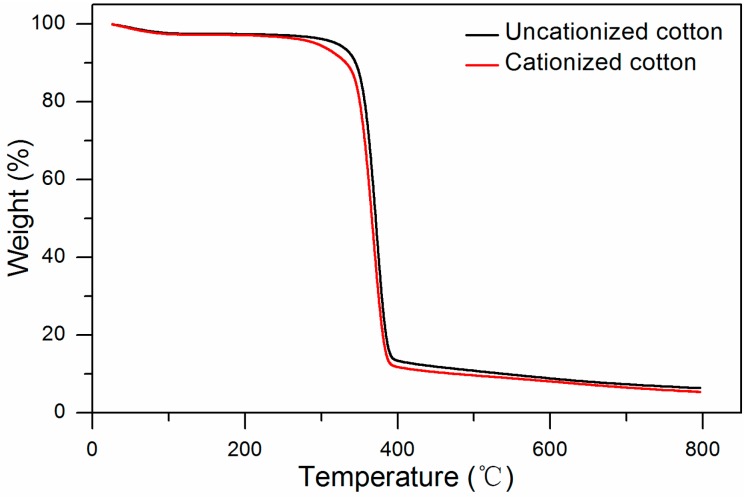
Thermal degradation of the uncationized and cationized cotton.

Thermogravimetric analysis was used to investigate the thermal properties of different fibers. Figure 8 presents the thermal degradation properties of the uncationized and cationized cotton. The stability of the uncationized cotton was slightly better than the cotton treated with GTA as a significant weight loss happened at 320 °C for uncationized cotton, while at 305 °C for the cationized one. This difference may be due to the grafting reaction between cotton fibers and GTA. The internal structure of the cationized cotton fibers becomes loose compared to the uncationized ones, and the crystallinity of the cellulose is reduced [29]. All cotton samples began to produce char at higher temperature [30,31].

### 2.4. Comparison of F% on Cationized and Uncationized Cotton

**Figure 9:**
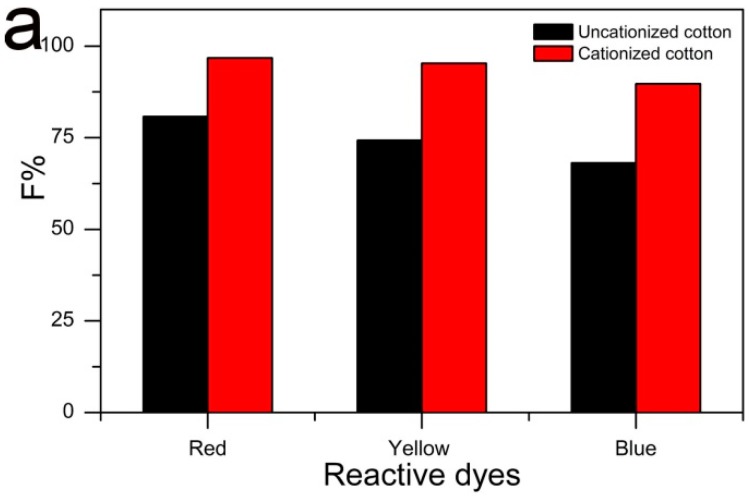

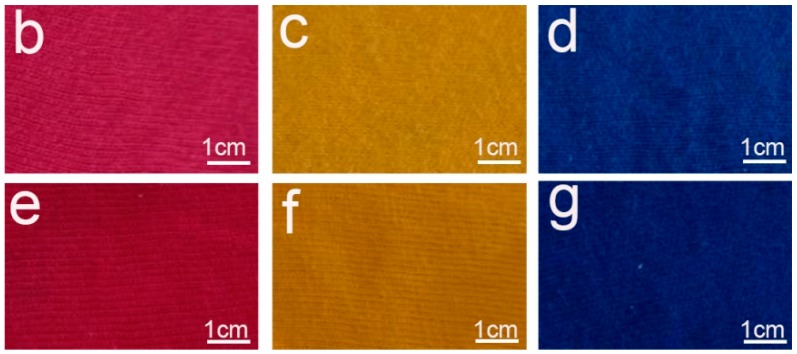
F% of C. I. Reactive Red 195, C. I. Reactive Yellow 145, and C. I. Reactive Blue 19 on the uncationized and cationized cotton fabrics (**a**) and digital pictures of C. I. Reactive Red 195, C. I. Reactive Yellow 145 and C. I. Reactive Blue 19 on uncationized fabrics (**b**–**d**) and cationized ones (**e**–**g**).

Figure 9a shows F% of C. I. Reactive Red 195, C. I. Reactive Yellow 145, and C. I. Reactive Blue 19 on both the cationized (prepared under the optimal conditions) and the uncationized cotton only being scoured and bleached. The digital pictures of two kinds of cotton that were colored with the above three reactive dyes are presented in Figure 9, as well. Due to the introduction of the cationic groups to cotton, reactive dyes are easily adsorbed on the cationic cotton without the addition of sodium sulfate or sodium chloride in the dyebath. A comparison of the F% results for dyeing the cationic cotton with that for dyeing the uncationized one in the presence of 60 g/L sodium sulfate, it could be seen that in all the cases, F% of the dyeings obtained on the former was much higher than that on the latter. F% was 80.79%, 74.26%, and 68.09 for uncationized cotton dyed with C. I. Reactive Red 195, C. I. Reactive Yellow 145, and C. I. Reactive Blue 19, respectively; while, F% was improved to 96.74%, 95.29%, and 89.72% on cationic cotton, respectively. These results indicated that with the cationic cotton prepared under the optimized conditions, dye utilization efficiency was enhanced greatly and savings in both dye and salt consumption were achieved in the designed process. The difference between the dyed samples can be observed from Figure 9. The colors of the cationized cotton fabrics pretreated with the combinative process all deeper than that of uncationized ones pretreated with only scoured and bleached steps.

### 2.5. Color Strength and Colorfastness Properties

Table 2 shows that the color strength (K/S) and colorfastness properties of the dyed fabrics in both salt-free and conventional dyeing process. K/S values of all three reactive dyes in salt-free dyeing were higher. K/S values of C. I. Reactive Red 195, C. I. Reactive Yellow 145, and C. I. Reactive Blue 19 were 13.0, 5.3 and 15.5 on uncationized cotton with addition of 60 g/L sodium sulfate. These results increased to 14.7, 7.2 and 18.5, respectively, when cationic cotton was dyed in the absence of salt. The wash fastness of the dyeing on the cationic cotton was all good, change of shade and staining of adjacent fabrics both being almost equal to the values obtained on the uncationized cotton. Dry and wet fastness of the dyes on the cationic fabrics was also comparable with that obtained from conventional dyeing. Moreover, light fastness testing of the dyeing on the cationic fabrics showed satisfactory results compared with that in the conventional dyeing. The above results indicated that the combinative pretreatment had no adverse effect on fastness properties of the tested reactive dyes.

### 2.6. Determination of Dye Penetrability

**Figure 10:**
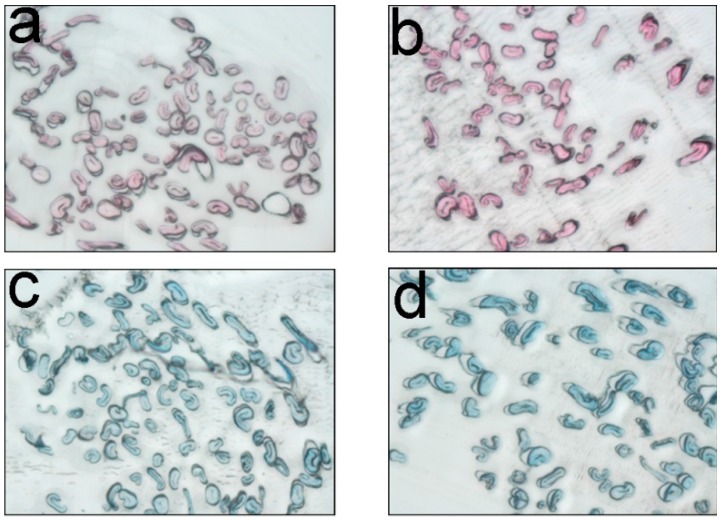
Cross-sections of uncationized (**a**,**c**) and cationized (**b**,**d**) cotton fibers dyed with C. I. Reactive Red 195 and C. I. Reactive Blue 19.

Dye penetrability commonly influences dye fixation and colorfastness properties. If dyes can penetrate into fibers well, it is beneficial for the reaction of the dyes with the fibers to achieve a high dye fixation. In the study, dye penetrability was tested with a light microscope and the results are shown in Figure 10. The figure shows that both C. I. Reactive Red 195 and C. I. Reactive Blue 19 can penetrate and coloring the inner parts of both the uncationized and cationized cotton fibers. The permeability of the cationized cotton was even better than that only being scoured and bleached. As GTA is a small cationic agent and cationization proceeds under alkaline condition and high-temperature of 90 °C, all these benefit the penetration of GTA in the fibers, which further benefits the penetration of the dyes. Moreover, it is obvious that the color of the inner parts of the cationic fibers in Figure 10b,d is much deeper than that of the uncationized ones in Figure 10a,c because the color yield of dyes on the cationic fibers is much higher.

## 3. Materials and Methods

### 3.1. Materials

Greige knitted cotton fabric (168 g/m^2^), glycidyltrimethylammonium chloride, scouring agent, hydrogen peroxide (30 wt %), sodium hydroxide, sodium sulfate, and anhydrous sodium carbonate were purchased from Tianjin Bodi Chemical Co., Ltd., Tianjin, China. Three reactive dyes used in this study are C. I. Reactive Red 195, C. I. Reactive Blue 19, and C. I. Reactive Yellow 145, and they are purchased from Zhejiang Shunlong Chemical Co., Ltd., Shaoxing, Zhejiang, China.

### 3.2. Combinative Pretreatment of Scouring, Bleaching, and Cationization

Exhaust method was employed for scouring, bleaching and cationizing greige cotton fabric. The experimental technique was adopted as follows: a greige cotton fabric sample was immersed in aqueous solution containing 4 g/L scouring agent along with 6 g/L hydrogen peroxide at 90 °C for 60 min, and then GTA and sodium hydroxide were added in the bath and cationization was performed for a certain time under different temperatures. Material to liquor ratio was adjusted to 1:20. The samples were then washed several times with boiling water and tap water to remove the impurities.

### 3.3. Dyeing Procedures

All dyeings were carried out in an XW-PDR laboratory dyeing machine with 12 shaking baths and a temperature and time control unit using a liquor-to-goods ratio of 20:1. The dye applied was 2% (o.w.f) for C. I. Reactive Red 195, 3% (o.w.f) for C. I. Reactive Blue 19, and 1% (o.w.f) for C. I. Reactive Yellow 145. Dyebaths were prepared by dissolving the dye in distilled water and both the uncationized and cationic fabric samples were immersed in the dyebaths at 30 °C. For conventional dyeing of the uncationized cotton, 60 g/L of anhydrous sodium sulfate was added to the dyebaths. The dyeing procedure for cationic cotton was chosen to allow dyeing in the absence of electrolyte. After dyeing at 30 °C for 30 min, the temperature was gradually increased to 60 °C at 2 °C/min, then 10 g/L sodium carbonate was added in the bath for dye fixation of 40 min.

After dyeing, the cotton fabrics were removed from the dyebaths and rinsed thoroughly in tap water. The rinse was collected for measurement of dye exhaustion. Then the dyed fabrics were subjected to boiling in a solution containing 0.2 g/L anionic detergent LS (Shanghai Auxiliary Co., Shanghai, China) at a liquor-to-goods ratio of 50:1 for 15 min until no dye was removed off, and then rinsed with water and allowed to air dry.

### 3.4. Whiteness Test

The degree of whiteness was measured with UltraScan XE Color Measuring and Matching Meter (HunterLab Co., Reston, VA, USA) by using the CIE method according to EN ISO 105-J02:1997 (E) standard [32,33].

### 3.5. Water Absorptivity and Diffusion Time

The water absorptivity and diffusion time of cotton was tested according to GBT 21655.1-2008.

### 3.6. Capillarity Effect Test

The capillarity effect of cotton fibers was tested according to FZ/T 01071-2008. The higher the wicking height is, the better the absorption and moisture.

### 3.7. Determination of Nitrogen Content and Cationization Degree

The nitrogen content of the cationic cotton was determined in triplicate by the Kjeldahl method. The samples were dried under vacuum at the temperature of 50 °C before measurement. For each condition, nitrogen content of three samples were measured to take average. In this study, the nitrogen content of the cationic cotton obtained under the optimal conditions was 0.0839%. The amount of modifier grafted onto the cotton fiber can be determined by the nitrogen content. Calculation method of cationization degree is according to the following formula (Equation (1)):

(1) $$\mathrm{Cationization}\;\mathrm{degree}\;\left(\mathrm{mmol}/g\right)=\frac{N\%\times 1000}{14.01}÷100$$

wherein, 14.01 is the relative atomic mass of nitrogen atoms, and N% is the nitrogen content (mass percentage) of the modified cotton fibers.

The cationization degree of modified cotton fibers is 0.0599 mmol/g under optimal conditions, which shows the degree of GTA grafted onto cotton fibers.

### 3.8. X-ray Diffraction

The X-ray diffraction (XRD) patterns of the fibers were measured stepwise in 2 h between 0° and 55° by a Rigaku diffractometer D/max-2400 (Rigaku, Tokyo, Japan). A monochromatic (graphite monochromator) Cu-Ka1-radiation (40 kV, 100 mA) was used.

### 3.9. Scanning Electronic Microscope

The surface morphology of the pretreated fibers was investigated using a NOVA NanoSEM 450 scanning electron microscope (SEM) (FEI Instruments Company, Hillsboro, OR, USA). The cotton fiber samples were coated with gold sputtering at room temperature before measurement.

### 3.10. Thermogravimetric Analysis

The thermal degradation property of the samples was performed on a Q500 thermoanalyzer from the TA instrument company (New Castle, DE, USA). A certain weight of sample was tested under N_2_ at a heating rate of 10 °C/min with the temperature ranging from 25 °C to 800 °C.

#### 3.10.1. Color Strength (K/S) and Dye Fixation (F%) Measurement

The color strength expressed as K/S value was calculated from Kubelka-Munk equation as shown below (Equation (2)):

K/S = (1 − R)^2^/2R (2)

The reflectance R of the dyed sample was determined on UltraScan XE Color Measuring and Matching Meter (HunterLab Co., Reston, VA, USA) at the wavelength of the minimum reflectance (the maximum absorbance) of each dyestuff.

Dyebath fixation (F%) of both the uncationized and cationized cotton was measured by sampling the dyebath before and after dyeing and soaping processes using Equation (3):

F% = (A_0_ − A_1_ − A_2_)/A_0_ × 100 (3)

where A_0_ is the absorbance of the dyebath before dyeing, and A_1_ andA_2_ are the absorbance of the dyebath after dyeing and that of the soaping bath, the absorbance was measured at the maximum absorption wavelength of each dye using HP 8453 UV–VIS spectrophotometer (Hewlett-Packard Development Co. Ltd., Palo Alto, CA, USA).

#### 3.10.2. Colorfastness Testing

The wash fastness of the reactive prints on cotton was tested according to AATCC Test Method 61-2001 with an S-1002 two-bath dyeing and testing apparatus (Roaches Co., West Yorkshire, UK). The crock fastness was tested according to AATCC Test Method 8-2001 using Y(B)571-II crockmeter (Darong Standard Textile Apparatus Co. Ltd., Wenzhou, China). The light fastness was tested according to AATCC Test Method 16-2001 using YG(B)611-V lightfastness tester (Darong Standard Textile Apparatus Co. Ltd., Wenzhou, China).

#### 3.10.3. Microscopic Analysis

Uncationized and cationized cotton fibers dyed with C. I. Reactive Red 195 and C. I. Reactive Blue 19 were used to carry out to the microscopic analysis. Cross-sections of the dyed cotton fibers were prepared using a LEICAEM UC6 microtome (Leica, Wetzlar, Germany). Images of the cross-sections were obtained at 600× magnification using an Olympus BX63 light microscope (Olympus Corporation, Tokyo, Japan).

## 4. Conclusions

In this study, cationization of cotton fabrics is designed to combine with their scouring and bleaching pretreatment. With this designed combinative pretreatment, enhanced dye fixation on the fabrics is easily realized in the absence of inorganic salt. The alkaline and high-temperature conditions of scouring and bleaching steps are utilized to promote cationization of cotton with GTA, thus, this combinative pretreatment is much alkali-, energy- and time-saving process compared with the previously-reported separated one. The performance of the pretreated fabrics, including whiteness, capillary effect, water absorptivity, and diffusion time all meet the requirements in the national standard. XRD and SEM results showed cationization in this combinative pretreatment did not affect the crystallization and surface morphology of the cationic fibers as the extent of cationization was small under the selected cationization conditions. With the combinative pretreatment, F% of C. I. Reactive Red 195, C. I. Reactive Yellow 145 and C. I. Reactive Blue 19 on the cationic cotton reached 96.74%, 95.29% and 89.72%, respectively, which are 19.7%, 28.3% and 31.8% higher than that on the uncationized fabrics, respectively. The dye colorfastness was all satisfactory. So using the convenient and effective combinative pretreatment, not only environmental pollution from salt addition in reactive dyeing was eliminated, dye utilization was also greatly enhanced. Based on the above results, it can be concluded that the combinative pretreatment was suitable for preparation of cationic cotton for salt-free reactive dyeing to achieve both effluent reduction and satisfactory application properties.

## Figures and Tables

**Table 1:** Whiteness of cotton after scouring and bleaching for different time.

Time (min)	Whiteness
30	68.07
45	73.63
60	77.04
75	76.54

**Table 2:** Color strength and colorfastness properties of the reactive dyes on cotton fabrics.

Dye	Fabrics	K/S	Wash Fastness			Rub Fastness		Light Fastness
			Change	Staining		Dry	Wet	
				Cotton	Wool			
C. I. Reactive Red 195	Uncationized	13.0	5	4–5	4–5	4–5	3–4	3–4
	Cationized	14.7	4–5	4–5	4–5	4–5	3–4	4
C. I. Reactive Yellow 145	Uncationized	5.3	4–5	4–5	4–5	4–5	4–5	6–7
	Cationized	7.2	4–5	4–5	4–5	5	4–5	6–7
C. I. Reactive Blue 19	Uncationized	15.5	4–5	4–5	3	4–5	3–4	7
	Cationized	18.5	4–5	4	3	4–5	4	7
